# Material security, life history, and moralistic religions: A cross-cultural examination

**DOI:** 10.1371/journal.pone.0193856

**Published:** 2018-03-07

**Authors:** Benjamin Grant Purzycki, Cody T. Ross, Coren Apicella, Quentin D. Atkinson, Emma Cohen, Rita Anne McNamara, Aiyana K. Willard, Dimitris Xygalatas, Ara Norenzayan, Joseph Henrich

**Affiliations:** 1 Max Planck Institute for Evolutionary Anthropology, Leipzig, Germany; 2 University of Pennsylvania, Philadelphia, United States of America; 3 University of Auckland, Auckland, New Zealand; 4 Max Planck Institute for the Science of Human History, Jena, Germany; 5 University of Oxford, Oxford, United Kingdom; 6 Victoria University of Wellington, Wellington, New Zealand; 7 University of Connecticut, Storrs, United States of America; 8 University of British Columbia, Vancouver, Canada; 9 Harvard University, Cambridge, United States of America; Universiteit van Amsterdam, NETHERLANDS

## Abstract

Researchers have recently proposed that “moralistic” religions—those with moral doctrines, moralistic supernatural punishment, and lower emphasis on ritual—emerged as an effect of greater wealth and material security. One interpretation appeals to life history theory, predicting that individuals with “slow life history” strategies will be more attracted to moralistic traditions as a means to judge those with “fast life history” strategies. As we had reservations about the validity of this application of life history theory, we tested these predictions with a data set consisting of 592 individuals from eight diverse societies. Our sample includes individuals from a wide range of traditions, including world religions such as Buddhism, Hinduism and Christianity, but also local traditions rooted in beliefs in animism, ancestor worship, and worship of spirits associated with nature. We first test for the presence of associations between material security, years of formal education, and reproductive success. Consistent with popular life history predictions, we find evidence that material security and education are associated with reduced reproduction. Building on this, we then test whether or not these demographic factors predict the moral concern, punitiveness, attributed knowledge-breadth, and frequency of ritual devotions towards two deities in each society. Here, we find no reliable evidence of a relationship between number of children, material security, or formal education and the individual-level religious beliefs and behaviors. We conclude with a discussion of why life-history theory is an inadequate interpretation for the emergence of factors typifying the moralistic traditions.

## Introduction

Over the past few decades, numerous scholars have sought to explain the emergence and spread of “moralistic” religious traditions. These traditions are characterized as those that emphasize adherence to prosocial norms under the threat of punishment by knowledgeable deities explicitly concerned with how we treat each other. Many have assessed how socioecological variables such as societal complexity [1, 2], resource scarcity [3], and animal husbandry [4], can help explain the emergence of these “moralistic high gods.”

There is some evidence that commitment to moralistic gods—deities thought to be concerned with moral norms, particularly those claimed to bestow costs or benefits to people based on their moral actions—post-date critical thresholds of societal complexity, suggesting they developed and spread as a response to that complexity [2]. Normative beliefs in divine punishment and supernatural monitoring by these deities may function adaptively by reducing the costs of punishing others for misconduct [1], while further expanding and/or stabilizing the sphere of human cooperation [5, 6]. Moreover, an emerging secularization literature shows that among contemporary state societies, greater material security [7], economic equality [8], and education [9] predict less overall religiosity cross-nationally. In other words, the functions that moralistic religions traditionally served may have been co-opted and out-competed by effective secular institutions, thus diminishing the significance of the former.

As detailed below, some researchers [10, 11] have challenged this view by arguing that a widespread shift in affluence was the prime mover of the genesis and ubiquity of the moralistic and ascetic traditions. According to this alternative theory, religious traditions turn moralistic not because widely shared beliefs in supernatural deities contribute to stabilizing wider prosociality or carry adaptive externalities, but rather because life history strategies covary with environmental fluctuations [11] and this predicts a concomitant shift in religious expression. Before discussing this hypothesis in more detail, we first briefly review life history theory.

Generally, mathematical models in life history theory are grounded on the idea that natural selection favors developmental and reproductive strategies that increase fitness, and that optimal life history strategies may co-vary with mortality regimes [12, 13] and ecological conditions, such as average resource abundance and the associated variance in realizable resources [14, 15]. With respect to empirical studies of human reproduction, life history theory is rendered more complicated by human flexibility and the demographic transition [16–21]. One commonly cited subset of the theory is framed in terms of reproductive speed (i.e., a “slow” vs. “fast” life history) where reproductive rate is predicted to co-vary with resource security.

There is some evidence regarding a positive relationship between material security and the number of children people have in traditional societies [22]. In post-demographic transition contexts, however, some evidence suggests that more educated parents will invest more time and resources in children [19]. However, increased education and material security are frequently associated with *lower* fertility rates [23, 24]. Foregoing reproduction in such contexts can both increase the socioeconomic status of subsequent generations, as well as reduce the number of children people have [25]. Further complications abound regarding how this “slow-fast” spectrum follows from evolutionary life history theory (see Discussion).

Aiming to extend these ideas to the domain of human cultural variation, Baumard et al. [10, 11, 26] recently argued that the rise of moralistic religious traditions (i.e., those with doctrines including something akin to the “Golden Rule”) and the decline of ritual devotion are indicative of slow life history strategies. Contrary to recent findings of the relationship between morally concerned deities and ecological duress [3] or social complexity [2], this work argues that moralizing religious beliefs are “a set of beliefs that are pragmatically held by slow-life individuals to help them moralize fast-life behaviours” [10]. In other words, wealthier people with fewer, “higher-quality” children are more prone to morally judge poorer people with relatively more children. This research predicts that at the individual level, “moralizing beliefs are more strongly held by people pursuing a slow strategy” [10, p. 4].

In an empirical study, Baumard et al. [11] found that greater projected energy capture (per capita/diem kcal, estimated from historical data) predicted the emergence of moralistic religions of the so-called “Axial Age,” a period (roughly between 800 and 200 BCE) often touted as a radical shift in human thought, religion, and social complexity [27, 28]. Though the details regarding how this might have happened remain unclear, the authors suggest that their results could be interpreted with life history theory (as expressed by the slow-fast continuum) insofar as “a massive increase in prosperity and certainty during the Axial Age may have triggered a drastic change in strategies, shifting motivations away from materialistic goals … toward long-term investment in reciprocation.” This “shift in priorities progressively would have impacted religious … traditions through a transmission bias, in favor of doctrines and institutions that coincided with the new values” [11, pp. 12-13]. Affluence moves “individuals away from ‘fast life’ strategies (resource acquisition and coercive interactions) and toward ‘slow life’ strategies (self-control techniques and cooperative interactions) typically found in axial movements” (p. 11). These “doctrines and institutions” associated with “slow-life strategies” include: increased asceticism, level of moral concern, punitiveness, and the breadth of knowledge (i.e., omniscience indicative of universalizing governance) attributed to deities, as well as the purported shift in emphasis from the ritual performances held by more traditional societies to one of “ethical commands” [10, 11].

There are a few limitations to and problems with these assertions and empirical tests. First, many of the features of religions often claimed to have developed during the “Axial Age” predate this period [6]. Second, as we detail in the Discussion section, evolutionary life history theory does not, in fact, predict a generalized “slow-fast” tradeoff [14, 15]. Rather, formal models of life history evolution demonstrate that the relationship between environmental harshness and life history variables is contingent on critical exogenous demographic and cultural factors.

Third, while the theory [10] details individual-level phenomena—the appropriate level of measurement for tests of life history processes—the test [11] uses coarse group-level wealth measures with no consideration of within-tradition variation. To their credit, Baumard et al. acknowledge that their measure “does not take into account the distribution of resources within a given society…[e.g., being] upper middle class in Greece…was probably much greater than what was available to the corresponding class in Persia or Egypt” (12). Here, they acknowledge that group-level per capita/diem kcal is an unreliable index of individual-level wealth distributions within groups. More specifically, group-level point estimates (e.g., mean kcals) are not indicative of within-group variation necessary to adequately test the life history predictions linking wealth and belief in more moralizing deities within populations. In fact, this may give misleading results (see below and Section 6 in S1 Supporting Information). Variation in beliefs and practices faces the same problem; models of life history theory cannot easily speak to group-level abstractions like “Christianity,” “Buddhism,” or “Axial.” In summary, tests of the life history interpretations of group-level patterns require higher-resolution data. The present research report attempts to do just this.

If popular life history predictions hold, then material security and education should correspond to fewer children. Assuming these conditions hold, if the aforementioned core features of moralistic religious traditions emerged due to shifting levels of wealth and generalized well-being, then—within the context of one’s community—an individual’s material security should be positively associated with his or her beliefs about the: 1) the level of moral concern of their deities, 2) the extent of divine sanctioning of moral norm-violators, and 3) the level of knowledge or omniscience (supernatural monitoring) of their deities. Moreover, material security should be 4) negatively associated with ritual participation. If these qualities attributed to deities emerge as a function of material security, we should also see a spike in the attribution of these qualities to locally salient—but relatively less moralistic, punitive, and knowledgeable—deities when resource security is higher. Here, we test these hypotheses using individual-level data collected in eight diverse field sites, using a modeling framework that accounts for variation within and across cultural groups.

## Method

### Participants

We recruited participants (*N* = 592; 311 females; mean age = 37.40, *SD* of age = 14.97; mean number of children (given birth to or fathered) = 2.45, *SD* of number of children = 2.43) from eight different field sites: (1) Coastal and (2) Inland Tanna, Vanuatu; (3) Lovu and (4) Yasawa, Fiji; (5) Porte aux Piment, Mauritius; (6) Kyzyl, Tyva Republic; (7) Hadzaland, Tanzania; and (8) Marajó, Brazil. Table 1 highlights some demographic and religious features of our sample and further descriptive statistics for each site are in Tables A-B in S1 Supporting Information. Across sites, participants gave at minimum, their verbal consent to participate after hearing a statement about informed consent approved by all researchers’ home institutions at the time of project execution.

**Table 1:** Descriptive statistics of each field site.

Site/Sample	World Religion	Economy	*N*	Children	Years Educ.	Food Sec.
Coastal Tanna	Christianity	Horticulture/Market	44	2.52 (1.86)	8.18 (3.55)	0.82 (0.39)
Hadza	None	Foraging	68	4.28 (2.61)	1.38 (2.68)	0.15 (0.36)
Inland Tanna	None	Horticulture	76	3.67 (3.53)	0.63 (2.08)	0.72 (0.45)
Lovu, Fiji	Hinduism	Market	76	2.24 (1.59)	8.77 (3.78)	0.14 (0.35)
Mauritians	Hinduism	Market	95	1.40 (1.58)	8.14 (2.98)	0.65 (0.48)
Marajó Brazilians	Christianity	Market	77	2.18 (2.56)	8.00 (3.53)	0.10 (0.31)
Tyva Republic	Buddhism	Herding/Market	81	1.70 (1.43)	15.44 (2.29)	0.72 (0.45)
Yasawa, Fiji	Christianity	Horticulture/Market	75	2.00 (2.07)	9.66 (2.42)	0.41 (0.50)
Grand M (SD)	—	—	74.00 (14.36)	2.45 (2.43)	7.63 (5.37)	0.46 (0.50)

Values are means and (standard deviations) for number of children, years of formal education, and food security. See Tables A-B in S1 Supporting Information for additional summary statistics.

Our participants vary in their adherence to world religious traditions including Christianity, Hinduism, and Buddhism, and also engage in a wide range of traditional religious practices including shamanism, animism, and ancestor worship. This sample is well-suited to test the target predictions insofar as it consists of individual-level data that allows us to assess relative material security within-and across sites that vary in their adherence to various traditions, and have locally specific distributions of all target variables.

### Materials and methods

The present data are from the publicly available Evolution of Religion and Morality Project dataset Version 3.0 [29]. We developed this cross-cultural dataset to test predictions about religious beliefs, behaviors, and cooperation. For the present study, our target variable types are: demographic measures, material security, and religious commitment. Further details of our measures can be found in Section 3 of the S1 Supporting Information, along with all data and scripts required to implement a complete reproduction of the analyses. All materials for a full reproduction of our analyses are available at https://github.com/bgpurzycki/Material-Security-and-Moralistic-Religions.

#### Demographics

We include sex and age as covariates in all regression models. In our models predicting number of children, we hold its obvious relationship with age constant. In order to account for the nonlinear relationship between age and number of children, and to minimize problems associated with multicollinearity, we include age as an exposure variable (i.e., removing pre- and post-reproductive windows), with the coefficient representing the elasticity of reproduction with respect to age (Section 3.1 in S1 Supporting Information).

We also asked for the total years of formal education completed by each respondent, and include this variable in some analyses to hold its potential relationship with achieved fertility outcomes [23, 24] and religious beliefs [30] constant. Finally, in order to hold constant the possible effects of increased family size on the outcomes of interest, we include number of children in some models of religious beliefs.

#### Material security

We measured material security using eight time-varying indices (Section 3.1 in S1 Supporting Information). In part due to our focus on current material security rather than perceived future prospects and partly due to the Hadza’s unfamiliarity with scales and similar time increments, we focus here on self-reported food security for the coming month: *Do you worry that in the next month your household will have a time when it is not able to buy or produce enough food to eat?* This measure was strongly correlated cross-culturally with other self-report measures of material security [31]. Participants responded in a dichotomous fashion (yes = 1; no = 0) and we subsequently reverse-coded these data for the sake of clarity. For the sake of comparison, we include additional analyses (without the Hadza) that use comparable continuous-scale measurements of participants’ confidence in their access to food in the coming month reported in Table C in S1 Supporting Information.

#### Religious commitment

We operationalize the construct of practicing a more “moralistic” religion by measuring the degree to which individuals claim their deities care about punishing theft, deceit, and murder, how knowledgeable deities are thought to be, and how often people perform devotional rituals to these deities. This operationalization directly taps key elements in Baumard et al.’s proposals, and avoids using the crude typological classifications such as “Christian” or “Muslim” that most researchers acknowledge as underspecified.

Our religion measures derive from extensive ethnographic interviews about religious beliefs and practices (Section 3.3 in S1 Supporting Information). Drawing on these interviews, we selected two locally salient deities that were differentially attributed with moral concern. One god we asked about was the most locally salient moralistic deity. The other was a relatively less moralistic, but locally important supernatural agent [5]. To measure explicit beliefs, we crafted questions about each variable of interest as it applies to each of these two kinds of spiritual agents. From these responses, we created measures that assessed how individuals conceptualize the: 1) concerns about moral behavior, 2) propensity toward punishment, and 3) scope of knowledge (i.e., breadth of supernatural monitoring) expressed by their deities. Finally, we assessed 4) the frequency in which participants engage in ritual devotions to these deities.

#### Analyses

All analyses are implemented using multi-level Bayesian regression models, with outcome distributions that are appropriately tailored to the empirical data; reproductive outcomes are fit using negative binomial regressions, ordered categorical outcomes are fit using ordered logistic regressions, and interval constrained outcomes are fit using beta regressions (Section 4 in the S1 Supporting Information). For the main regression models, we focus on within-group variation by placing weak priors on the parameters controlling inter-site variation in intercepts and slopes (partial pooling). In Tables H-L in the S1 Supporting Information, we also report results for fully pooled models where inter-site variation was fixed at zero, strictly for the sake of illustration. All reported results include 90% equal tail posterior credibility intervals. For our predictions about religion, we assessed each deity in separate blocks; in the event that beliefs about moralistic deities influence or bleed into beliefs about local deities, we held corresponding moralistic deity variables constant in our local deity models. Model descriptions, results tables, diagnostics, additional analyses and links to analytical scripts are included in the S1 Supporting Information.

## Results

### Do materially secure individuals have fewer children?

**Fig 1:**
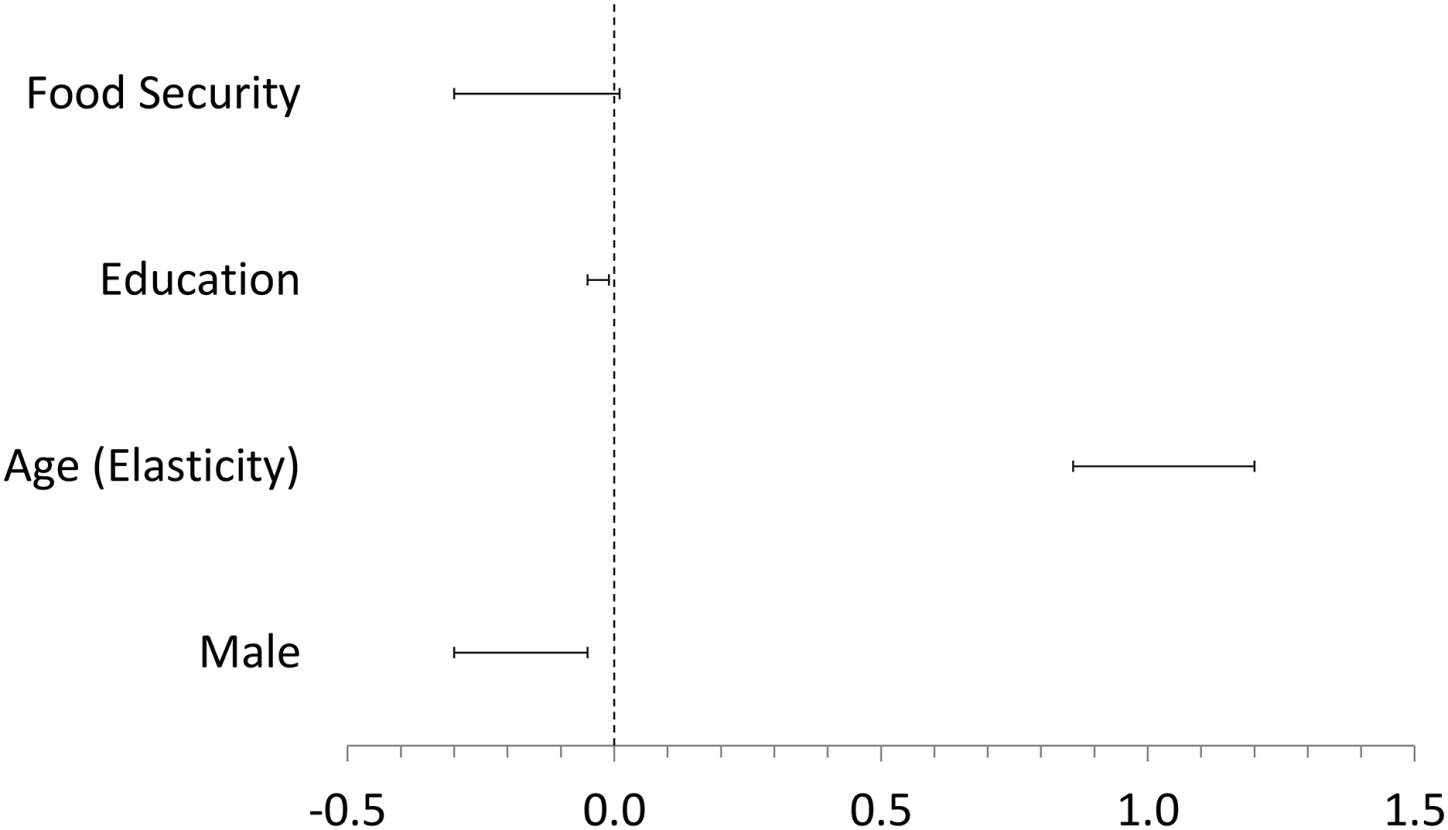
90% credibility intervals of mean estimates for factors predicting achieved fertility (Model 1 in Table 2). Effects to the right of zero are positive and effects to the left of zero are negative.

Table 2 presents three model specifications crafted for the purposes of assessing the robustness of the target variables. Model 1 represents the full model, illustrated in Fig 1. Holding age and sex constant, greater levels of material security are associated with fewer children across models. Additionally, years of formal education predict fewer children, a result consistent with previous work using data from European sources [23, 24]. Model 2 predicts that, on average across populations, being materially secure leads to a difference of -0.73 (-1.74, 0.08) children born for a woman who is past child-bearing years. Model 3 shows that an additional decade of schooling yields an average change of -1.58 (-3.29, -0.24) children born for a post-reproductive woman.

**Table 2:** Cross-population mean estimates of achieved fertility with 90% credibility intervals.

	Model 1	Model 2	Model 3
Food Security	-0.14	-0.13	—
	(-0.30, 0.01)	(-0.27, 0.01)	
Education	-0.03	—	-0.03
	(-0.05, -0.01)*		(-0.05, 0.00)*
Age (Elasticity)	1.02	1.07	1.01
	(0.86, 1.20)*	(0.90, 1.24)*	(0.83, 1.18)*
Male	-0.17	-0.19	-0.18
	(-0.30, -0.05)*	(-0.31, -0.08)*	(-0.30, -0.06)*
Intercept	-1.94	-2.31	-2.01
	(-2.55, -1.31)	(-2.88, -1.73)	(-2.64, -1.36)

Model 1 is the full model, and Models 2 and 3 drop education and food security outcomes, respectively. Across populations, we see proportionality between exposure time to risk of reproduction and number of children, as indicated by the elasticity estimate on age being centered on the value of 1. Males show reduced age-specific production of offspring relative to females. We note reliably negative average effects of education and wealth security on achieved fertility.

*Denotes credibility intervals that do not cross zero.

The qualitative results are robust to analysis using a fully pooled model. The supplementary code generates site-specific plots of the posterior estimates of regression coefficients. There, we demonstrate that the cross-population average estimates are similar to the results of each population (i.e., inter-site variation is small, and random effects are consistently on one side of zero).

### Do materially secure individuals conceptualize deities as more moralistic?

Recall that we selected two deities as targets for our questions precisely for the variation they exhibited in explicit association with moral concern. We modeled participants’ beliefs about moralistic *and* locally salient supernatural beings’ moral concern using the same set of covariates. If more materially secure respondents think of the relatively less moralistic local deities to be *more* moralistic than their materially insecure counterparts, this would provide an even better test of the driving hypothesis. In other words, we should be able to detect the emergence of moralistic gods when material security is higher, both within and across sites.

**Fig 2:**
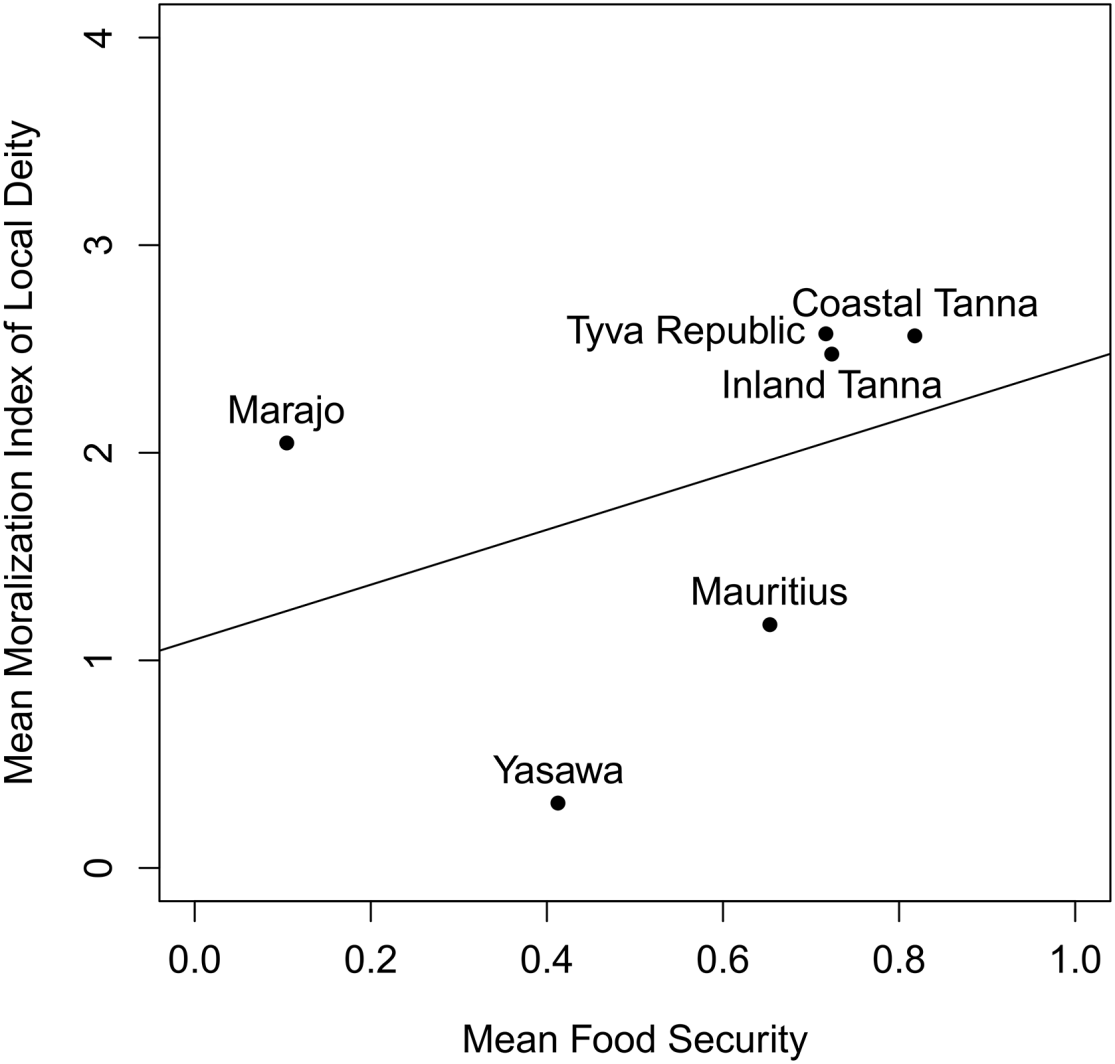
Group-level moralization of local deities appears to increase as a function of group-level material security. Note that the Hadza are missing due to difficulty with scale items and the Lovu are missing due to a lack of local deity data. This figure illustrates how aggregate, group-level patterns can be misleading for individual-level inferences. Compare this to the null effects in the Local Deity block in Fig 2 and Table D in S1 Supporting Information.

Fig 2 assesses our data at a similar level of abstraction employed by [11]. It plots the group-level grand means of material security and the moral index for local deities. The regression line is a simple linear regression line where the intercept is 1.10 (90% cred. int. = -0.54, 2.74) and the slope is estimated at 1.32 (90% cred. int. = -1.31, 3.96). Though a poorly-specified model detailing an implausible relationship, it is in the predicted direction of Baumard et al.’s hypotheses; a groups’ average material security appears to be associated with the degree to which that group on average claims local deities care about morality. As within-site variation may, for example, be negative, such a result may be misleading.

**Fig 3:**
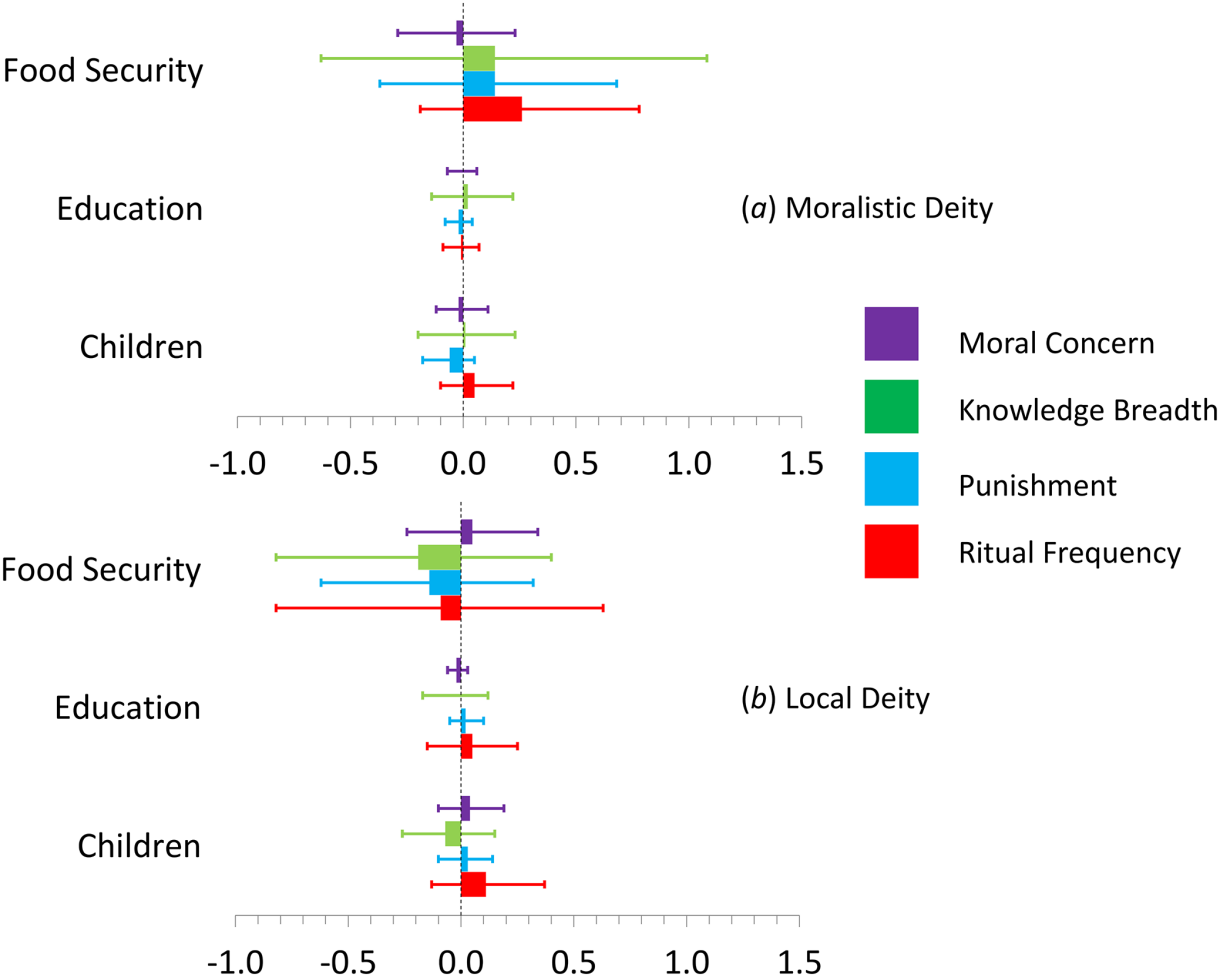
Mean estimates and 90% credibility intervals for the levels of moral concern, knowledge breadth, punishment, and self-reported devotional ritual frequency attributed to moralistic (*a*) and local (*b*) deities as a function of food security, years of formal education and number of children. These results hold participant sex and age constant. All values are from the results tables taken from the full models in Tables D-G in S1 Supporting Information. The end points of histograms are mean estimates. We include them for easier visual comparison of relative direction and distance from zero. Narrower error bars indicate more precise estimates. Effects to the right of zero are positive and effects to the left of zero are negative. Error bar symmetry around zero indicates no reliable effect; we found no evidence supporting any of the target predictions about religion.

In our models that fully account for within- and cross-site variation, the target demographic predictors failed to account for the moralization of gods’ concerns (Fig 3). In all of our models, only moralistic deities’ moral concern predited local deities’ attributed moral concerns. These findings reveal no relationship between material security and belief in more moralistic local deities within any given community, but there may be site-level covariance in the frequency of material security and the likelihood of respondents claiming that local deities care about moral behavior (see code). While the evolutionary dynamics proposed by Baumard et al. [10, 11, 26] are best evaluated at the level of individuals clustered by site (as we do here), a deeper analysis of the evolutionary dynamics underlying the weak group level covariance uncovered here with a larger sample of populations may be warranted.

Fig 3 illustrates the mean estimates for the target religious commitment variables for all full models. It shows that there is no evidence of a relationship between our demographic “life history” variables and our religion measures for either deity. To test whether or not material security accounts for the degree to which participants attribute moral concern to their deities, we regressed the moral indices on the aforementioned demographic variables. Contrary to the hypothesis that material security should predict greater moral concern attributed to deities, we found no such relationship; within sites, individuals living in more fortunate circumstances do not claim their deities care more about morality.

### Do materially secure individuals claim their deities know more?

We also assessed whether or not material security accounts for participants’ views of supernatural beings’ knowledge and monitoring. Again, we found no such relationship (green in Fig 2). Food security, sex, number of children, and years of formal education are not associated with how much people claim their deities know or monitor. How much participants claim the moralistic deities know, however, reliably predicted how much they claimed local deities know.

### Do materially secure individuals claim deities punish more?

Here, too, we found no relationship between food security and the claimed moralistic punishment of local and moralistic gods (blue in Fig 2). Food security, sex, number of children, and years of formal education are not associated with how much people claim their moralistic deities punish, but again, moralistic deities’ punishment scores had a strong association with local deities’ punishment scores.

### Do materially secure individuals participate in rituals less often?

To test whether or not material security predicts ritual practice frequency, we regressed self-reported devotional ritual frequency on the target demographic variables. We found no evidence suggesting that food security predicts less ritual performance (red in Fig 2). Note, however, that while the effect (0.26) is not reliable (90% cred. int. = -0.19, 0.78), ritual frequency toward moralistic gods is in the reverse of the predicted direction. For local deities, the only reliable relationship found is the positive one between how often participants engage in rituals for moralistic gods.

## Discussion

### Life history, material security, and reproductive outcomes

Recent work [10, 11, 26] has argued that models in life history theory, coupled with evidence of fluctuating state-transitions in environmentally-linked variables such as energy capture, affluence, or material security can explain the rise of moralistic religions. However, earlier [14] and more recent [15] advances in life history theory suggest the very opposite. Rather than absolute affluence having predictable effects on human motivation and reward systems or shifts from “slow” to “fast” life history strategies, these advances show that: 1) evolutionary theory does not, in general, predict that harsh environments (i.e., those with severe resource stress and high mortality rates) should favor fast life histories, or that affluent environments will favor slow life histories, and 2) that most life history models used to explain phenotypically plastic (i.e., cultural and behavioral) responses to fluctuating environmental states have been misapplied and/or draw misleading conclusions.

Although the prediction that harsh environments select for faster life histories is a popularly cited one among some anthropologists and evolutionary psychologists [32, 33], such hypotheses are typically presented with little formal, mathematical support [14, 15]. In some cases, evolutionary theory does predict that harsh environments should favor fast life histories, but this result does not hold in general, and the mechanistic details of the system—such as age-structure, parental investment, and how population dynamics are regulated—mediate which type of strategy will be favored in each type of environment [15].

Furthermore, the formal evolutionary models underpinning life history theory [15] typically assume that an entire population is genetically evolving toward a uniformly changing environment. Intuitively, we might assume that the optimal plastic response to a heterogeneous environment would be to employ the fixed behavior of a population that uniformly inhabits such conditions. However, as Baldini [15] demonstrates, this intuition is inconsistent with what the theory actually predicts; we require formal models of plastic life history strategies to make useful empirical predictions concerning how populations will flexibly adapt to specific kinds of fluctuating environments. The outcomes of adaptive strategies in fluctuating environments can be counterintuitive (e.g., food storage leading to more extreme famines; [34]), and strongly depend on the details of the population and environmental system [35].

This being said, we did find the same empirical trend using individual-level data that Baumard et al. [11] assume to exist. That is, increased material security is associated with decreased age-specific fertility. This also places our findings in line with a large but variable empirical literature finding such relationships in humans. However, it is not clear from our cross-sectional analysis if individuals with low food security have faster life histories and more children as an adaptive, risk-sensitive response to ecological circumstances, as some models might predict [36–38], or if more mundane reasons exist. For example, people with more children might be less certain about future food availability simply because they have more mouths to feed in their household [39]. Our results are consistent with either or both of these interpretations.

We also find a relationship between education and reproduction. It could be that the more time parents invest in their own education, the more likely they are to delay reproduction for non-adaptive reasons. Alternatively, there may be adaptive reasons for investment in the education and embodied capital of oneself and of one’s children [18, 23, 24, 40]. As is also the case with our findings concerning material security, the direction of causality here is ambiguous.

Despite this causal ambiguity concerning the correlates of material security, our cross-sectional analyses do demonstrate that our measures of food security and education can predict life history related outcomes cross-culturally; so our failure to find a relationship between material security and our measures of religious commitments is not easily dismissed on the basis of an ineffective operationalization of the material security measure.

### Moral religions and material security

Food security failed to account for the degree to which people claim their deities: 1) care about morality, 2) punish people, and 3) engage in supernatural monitoring. It also failed to account for: 4) respondents’ levels of ritual devotion to their deities. We found no support for any of the target predictions [10, 11], in either class of deities (moralistic or local), regardless of the other variables we included in models.

Of course, some key differences between our methods and those that others have employed may have contributed to these null results. While [11] used highly aggregated measures of projected energy capture for eight large geographic regions as a proxy variable for human affluence, we used individual-level, subjective measures of food security as an indicator of affluence. Additionally, Baumard et al. sampled traditions predetermined to be “moralistic” or “Axial” by academic consensus (which focuses only on elites), whereas we determined the degree to which individuals engaged in beliefs and behaviors typifying such traditions by eliciting and quantifying the characteristics they attributed to their deities.

While our data have the benefit of being grounded in individual sensibilities rather than coarse, group-level characterizations derived from historical sources involving substantial projections, they do reflect an ethnographic present that already includes the presence of these “moralistic” traditions, and do not assess their *de novo* emergence. Note again, however, that we saw no obvious association between food security and increased attributed moral concern to local deities. In other words, deities with relatively less moral concern do not appear to be evolving into moralistic deities due to any factor associated with material security. While our results have implications for the past, we do not assess data derived or postulated from the historical record.

Baumard et al. favor life-history theory as an explanation for the patterns they find [10, 11, 41]. Our analysis suggests that while the popular life history assumptions hold, they do not help to explain the target features of religion. While our results suggest that life history theory is not as useful as they might hope, longitudinal and detailed demographic, economic, and ethnographic data from multiple populations are crucial in order to reliably determine whether or not life history theory is helpful in explaining the emergence of the so-called “moralistic” traditions.

## Supporting information

S1 Supporting Information Descriptive demographic statistics, methods, statistical models, main and supplementary analyses. (PDF)
