# Supplementary material for: Material security, life history, and moralistic religions: A cross-cultural examination
Source: PLoS One. 2018 Mar 7;13(3):e0193856. doi: 10.1371/journal.pone.0193856 (PMC5841807; doi:10.1371/journal.pone.0193856)
Supplement: S1 Supporting Information — (PDF) [file pone.0193856.s001.pdf]

S1 Supporting Information for:
Material security, life history, and moralistic religions: A cross-cultural examination

Benjamin Grant Purzycki[1]*, Cody T. Ross[1], Coren Apicella[2], Quentin D. Atkinson[3,4], Emma Cohen[5], Rita Anne McNamara[6], Aiyana K. Willard[5], Dimitris Xygalatas[7], Ara Norenzayan[8], and Joseph Henrich[9]

1 Max Planck Institute for Evolutionary Anthropology, Leipzig, Germany
2 University of Pennsylvania, Philadelphia, United States of America
3 University of Auckland, Auckland, New Zealand
4 Max Planck Institute for the Science of Human History, Jena, Germany
5 University of Oxford, Oxford, United Kingdom
6 Victoria University of Wellington, Wellington, New Zealand
7 University of Connecticut, Storrs, United States of America
8 University of British Columbia, Vancouver, Canada
9 Harvard University, Cambridge, United States of America

*benjamin_purzycki@eva.mpg.de

Contents

1 Introduction **2**

2 Descriptive statistics **2**

3 Methodological notes **2**
3.1 Demographics and material security 3
3.2 Deity selection 3
3.3 Religious commitment 4

4 Statistical models **5**
4.1 A model of reproduction 5
4.2 A model of moralizing deities 5
4.3 A model of omniscient deities 6
4.4 A model of supernatural punishment 6
4.5 A model of ritual frequency 6

5 Analyses **6**
5.1 Main analyses 7
5.2 Supplementary analyses 8

6 Discussion **9**

1 Introduction

In this supplement to the main report, we: (1) present the relevant descriptive statistics of our data set, (2) provide additional methodological notes, (3) describe the statistical models used in the main and supplementary analyses, and (4) present the results of the primary and supplementary analyses referred to in the main paper.

2 Descriptive statistics

Table A reports the descriptive demographic statistics of our samples. Table B reports the summary statistics for the target religion data for both the "moralistic" (M) and "local" (L) deities. These data include the: (1) moralization, (2) punishment, and (3) knowledge breadth measures of two gods, and (4) participants' self-reported frequency of engaging in rituals devoted to these two deities. We selected these gods from preliminary ethnographic interviews on the basis of one being the most locally salient god concerned with interpersonal norms with costs and benefits to others (i.e., "morality") and another less morally-concerned supernatural being, but with local significance.

Table A. Descriptive demographic statistics, mean (standard deviation). Updated from Supplementary Materials in [1].

Site/Sample	Sampling Method	*N*	Females	Age	Children	Formal Ed.	Food Sec.
Coastal Tanna	Cluster sampling (census)	44	23	35.02 (14.13)	2.52 (1.86)	8.18 (3.55)	0.82 (0.39)
Hadza	Entire camps	68	31	39.82 (12.08)	4.28 (2.61)	1.38 (2.68)	0.15 (0.36)
Inland Tanna	Entire community	76	38	37.00 (16.17)	3.67 (3.53)	0.63 (2.08)	0.72 (0.45)
Lovu, Fiji	Door-to-door	76	52	44.56 (16.94)	2.24 (1.59)	8.77 (3.78)	0.14 (0.35)
Mauritius	Random (street) sampling	95	28	36.92 (15.36)	1.40 (1.58)	8.14 (2.98)	0.65 (0.48)
Marajó Brazilians	Random sampling (census)	77	40	34.12 (13.08)	2.18 (2.56)	8.00 (3.53)	0.10 (0.31)
Tyva Republic	Random/chain sampling (street)	81	58	33.53 (12.52)	1.70 (1.43)	15.44 (2.29)	0.72 (0.45)
Yasawa, Fiji	Door-to-door	75	41	38.04 (15.91)	2.00 (2.07)	9.66 (2.42)	0.41 (0.50)
Grand Mean	–	74.00	38.88	37.40	2.45	7.63	0.46
(SD)	–	(14.36)	(11.81)	(14.97)	(2.43)	(5.37)	(0.50)

Table B. Decriptive religiosity statistics for both moralistic (M) and local (L) deities, mean (standard deviation). Variables include deities' punishment (Punish), knowledge breadth (Know), and moral concern (Moral) ratings as well as ritual frequency (Ritual). Updated from Supplementary Materials in [1].

Site/Sample	Punish (M)	Punish (L)	Know (M)	Know (L)	Moral (M)	Moral (L)	Ritual (M)	Ritual (L)
Coastal Tanna	0.82 (0.29)	0.48 (0.23)	1.00 (0.00)	0.52 (0.39)	2.74 (1.20)	2.56 (0.99)	3.55 (0.55)	2.09 (1.04)
Hadza	0.68 (0.45)	0.56 (0.45)	0.76 (0.41)	0.72 (0.44)	–	–	–	–
Inland Tanna	0.67 (0.35)	0.67 (0.31)	0.93 (0.25)	0.80 (0.37)	2.73 (1.32)	2.47 (1.37)	3.65 (0.84)	3.09 (1.29)
Lovu, Fiji	0.84 (0.26)	–	0.98 (0.10)	–	3.13 (0.77)	–	1.30 (0.74)	–
Mauritius	0.64 (0.38)	0.43 (0.39)	0.91 (0.25)	0.48 (0.45)	1.98 (1.02)	1.17 (1.05)	2.77 (1.59)	0.65 (1.30)
Marajó	0.79 (0.32)	0.57 (0.41)	0.97 (0.16)	0.80 (0.39)	2.40 (1.09)	2.05 (1.13)	3.44 (0.88)	3.04 (1.02)
Tyva Republic	0.73 (0.33)	0.74 (0.31)	0.86 (0.31)	0.78 (0.33)	2.66 (1.13)	2.57 (1.12)	2.58 (1.06)	1.73 (1.16)
Yasawa, Fiji	0.55 (0.15)	0.48 (0.10)	1.00 (0.00)	0.00 (0.00)	3.78 (0.56)	0.31 (0.13)	1.11 (0.55)	0.00 (0.00)
Overall	0.71 (0.33)	0.57 (0.35)	0.93 (0.23)	0.57 (0.46)	2.75 (1.16)	1.72 (1.34)	2.51 (1.39)	1.50 (1.62)

3 Methodological notes

The data used for this report are from the publicly available [1, 2] Evolution of Religion and Morality Data Set (Version 3.0). All original methods protocols are available at: `https://github.com/bgpurzycki/Evolution-of-Religion-and-Morality`. All data, R scripts needed to replicate our analyses, and methods for rescaling all necessary

variables are available here: `https://github.com/bgpurzycki/Material-Security-and-Moralistic-Religions`.

Note that as the Indo-Fijian sample (Lovu) contributed no local deity data, all local deity models do not include this sub-sample. Additionally, because the Hadza had difficulty with scales, all analyses assessing the moral concern of deities, and devotional ritual frequency do not include them.

3.1 Demographics and material security

To address the nonlinear relationship between age and reproductive success, we converted age into an exposure variable by subtracting 15 from each age value. Then, we converted all exposure measures >45 to 45 (i.e., we do not count years lived in the post-reproductive period as contributing to the risk of reproductive success) [3].

The material security measure we employed here was highly correlated ($r = 0.98$) with "event security," which is the "ability to fund major household events, including illness, weddings, births, funeral" elsewhere [4]. This therefore lends itself for use as a general material insecurity measure. In addition to food insecurity, we also asked about confidence in food acquisition: *How certain are you that you will be able to buy or produce enough food to eat in the next* ___________ on a 5-point Likert scale from -2 to 2; very uncertain (-2), a little uncertain (-1), I don't know (0), a little certain (1), and very certain (2). There were four time variations of these questions: (a) month, (b) six months, (c) year, and (d) five years.

This worked well in all of our populations except among Hadza foragers, who do not keep track of dates in this fashion. There, only questions regarding month and year were asked. Moreover, Hadza participants had difficulty with continuous scales rendering the second set of questions difficult. Additionally, mean material insecurity and confidence over these time frames had a moderate negative correlation (r = -0.41, $P \leq 0.001$). We therefore focus on present food insecurity in the main text in order to avoid issues associated with multicollinearity, to maximize the sample size, and to keep our measures consistent across field sites.

However, as reported in Table C, we reproduced the same models with present material confidence without the Hadza sample. As the original data for material confidence was on a scale of -2 to 2, we rescaled the data. Values <1 were recoded with zeroes (i.e., a lack of certainty or confidence) whereas values >0 were recoded with ones (i.e., some certainty in food procurement over the next month). All other procedures were identical to those resulting in Table 1 of the main text. There were no differences in qualitative results.

3.2 Deity selection

In each site other than Lovu, Fiji, we asked participants about two locally salient deities. These were selected based on preliminary ethnographic interviews (the "Religious Landscape Interview"; see protocols). In these interviews, we asked participants (from separate samples when available) to freely-list locally important deities and spirits. Once listed, we then asked participants to respond to the following questions about each listed deity: (1) *Is* ___________ *concerned about what people do or how they behave?*; (2) *Can* ___________ *see into people minds or know their thoughts and feelings?*; (3) *Does* ___________ *punish people who behave in ways that* ___________ *does not like?*; and (4) *Does* ___________ *reward people who behave in ways that* ___________ *likes?* We then selected one frequently-listed deity in each site with the highest values for these questions (where "yes" = 1 and "no" = 0) and one locally important deity with a relatively lower composite rating for these items to inform the design of the main data collection procedure.

Table C. Cross-population mean estimates of reproductive success outcomes with 90% credibility intervals using material confidence (Hadza sample not included). Model 1 is the full model, and Models 2 and 3 drop education and food confidence outcomes, respectively. Across populations, we see proportionality between exposure time to risk of reproduction and reproductive success, as indicated by the elasticity estimate on age being centered on the value of 1. Males show reduced age-specific production of offspring relative to females. We note reliably negative average effects of education and food confidence on reproductive success. *Denotes credibility intervals that do not cross zero.

	Model 1	Model 2	Model 3
Material Confidence	-0.11	-0.11	—
	(-0.26, 0.04)	(-0.21, -0.01)*	
Education	-0.03	—	-0.03
	(-0.05, 0.00)*		(-0.05, 0.00)*
Age (Elasticity)	1.08	1.18	1.07
	(0.89, 1.26)*	(1.01, 1.39)*	(0.92, 1.22)*
Male	-0.20	-0.23	-0.22
	(-0.33, -0.06)*	(-0.35, -0.06)*	(-0.35, -0.08)*
Intercept	-2.17	-2.52	-2.20
	(-2.75, -1.48)*	(-3.04, -2.07)*	(-2.80, -1.62)*

3.3 Religious commitment

We examined three target features of respondents' conceptualizations of their deities: gods' moral concern, punishment propensities, and attributed knowledge breadth. Further analyses are available in the Supplementary Materials in [1]. Our "moral index" of deities' concern consists of the mean value of three questions: *How important is it for ___________ to punish ...* (a) theft, (b) lying, and (c) murder. Responses were on scales of 0 to 4: (0) Not important at all; (1) A little important; (2) Important; (3) Very important; (4) The most important.

The items for the moral index for the moralistic ($\alpha = 0.87$, 95% CI [0.85, 0.89]) and local ($\alpha = 0.89$, 95% CI [0.88, 0.91]) deities were highly inter-correlated. In comparing these scales using a two-tailed paired Wilcoxon signed rank test (V = 38003, P <0.001), the moralistic deities (M = 2.76, SD = 1.16) were indeed rated as more morally concerned than the local deities (M = 1.72, SD = 1.34).

We measured punishment using ordered categories corresponding to the number of dichotomous questions (0 = no; 1 = yes) participants answered "yes" to: *Does ___________ ever punish people for their behavior?* and *Can ___________ influence what happens to people after they die?*

We measured the attributed breadth of deities' knowledge using ordered categories corresponding to the number of two other dichotomous questions they answered: *Can ___________ see into people's hearts or know their thoughts and feelings*? and *Can ___________ see what people are doing if they are far away in* [a distant town or city known to participants][1]?

As part of our protocols, we asked an indicator question: *Do you perform activities or practices to talk to, or appease ___________?* with the follow-up question asking how often on a frequency scale: (4) = Every day or multiple times per day; (3) = A few times per week; (2) = A few times per month; (1) = A few times per year; and (0) = Very rarely/never. We model responses to this frequency scale item as ordered categories.

[1]Note that one individual from Marajó answered this question for the local deity (St. Mary) by saying "Só se você acredita" (trans. only if you believe). We converted this into a missing value.

4 Statistical models

4.1 A model of reproduction

To model fertility outcomes, we declare that the reproductive outcomes of individual i, $R_{[i]}$, are distributed according to a negative binomial distribution, with mean, $\phi_{[i]}$, and inverse scale, B:

$$R_{[i]} \sim \text{Negative Binomial}(\phi_{[i]}B, B) \tag{1}$$

We use a log link function for ϕ, such that $\log(\phi_{[i]})$ is given by:

$$\begin{aligned}\log(\phi_{[i]}) = \beta_{[1,j]} + \beta_{[2,j]} \log(A_{[i]}) + \beta_{[3,j]} M_{[i]} \\ + \beta_{[4,j]} E_{[i]} + \beta_{[5,j]} S_{[i]}\end{aligned} \tag{2}$$

where $A_{[i]}$ is the shifted and truncated age of individual i (so that it measures years of reproductive exposure), $M_{[i]}$ is an indicator variable describing if individual i is male, $E_{[i]}$ is the education of individual i in years, and $S_{[i]}$ is a binary measure of food security.

We then use a multi-level model structure to partially pool information about the β parameters which are unique to each population, j:

$$\beta_{[j]} \sim \text{Multivariate Normal}(\mu, \Sigma) \tag{3}$$

where the covariance matrix Σ is defined as:

$$\Sigma = \text{Diag}(\sigma)\rho\text{Diag}(\sigma) \tag{4}$$

Here, σ gives the variance of the random effects and ρ gives the correlation matrix. In the main analysis, we use weak half-Cauchy priors on the elements of μ and σ:

$$\mu_{[d]} \sim \text{Normal}(0, 5) \tag{5}$$

$$\sigma_{[d]} \sim \text{Cauchy}(0, 2.5)T[0, \infty] \tag{6}$$

$$B \sim \text{Cauchy}(0, 2.5)T[0, \infty] \tag{7}$$

In the supplementary analyses—for this model and the models detailed below—we also use a fully pooled model that omits group structure. In other words, we set:

$$\beta_{[j]} = \mu \tag{8}$$

4.2 A model of moralizing deities

To model moralization outcomes, we declare that moralization outcomes provided by individual i, $Z_{[i]}$, after scaling are distributed according to a mean and dispersion parameterized Beta distribution, with mean, $\psi_{[i]}$, and dispersion, κ:

$$Z_{[i]} \sim \text{Beta}(\psi_{[i]}, \kappa) \tag{9}$$

We use a logit link function for ψ, such that $\text{logit}(\psi_{[i]})$ is given by:

$$\begin{aligned}\text{logit}(\psi_{[i]}) = \beta_{[1,j]} + \beta_{[2,j]} \log(A_{[i]}) + \beta_{[3,j]} M_{[i]} \\ + \beta_{[4,j]} E_{[i]} + \beta_{[5,j]} S_{[i]}\end{aligned} \tag{10}$$

and the prior on κ is:

$$\kappa \sim \text{Cauchy}(0, 2.5)T[0, \infty] \tag{11}$$

with all other values being comparable to the reproduction model.

4.3 A model of omniscient deities

To model gods' knowledge outcomes, we declare that knowledge outcomes provided by individual i, $K_{[i]}$, are ordered categories. As such, we model them using ordered categorical regression with a linear model for ξ, and a vector of random cut-points C:

$$K_{[i]} \sim \text{Ordered Categorical}(\xi_{[i]}, C) \tag{12}$$

We use an identity link function for ξ, such that $\xi_{[i]}$ is given by:

$$\begin{aligned}\xi_{[i]} = \beta_{[1,j]} + \beta_{[2,j]} \log(A_{[i]}) + \beta_{[3,j]} M_{[i]} \\ + \beta_{[4,j]} E_{[i]} + \beta_{[5,j]} S_{[i]}\end{aligned} \tag{13}$$

with all other values being comparable to the reproduction model.

4.4 A model of supernatural punishment

To model punishment outcomes, we declare that punishment outcomes provided by individual i, $P_{[i]}$, are ordered categories. As such, we model them using ordered categorical regression with a linear model for ξ, and a vector of random cut-points C:

$$P_{[i]} \sim \text{Ordered Categorical}(\xi_{[i]}, C) \tag{14}$$

with all other values being comparable to the omniscience model.

4.5 A model of ritual frequency

To model ritual outcomes, we declare that ritual outcomes provided by individual i, $Y_{[i]}$, are ordered categories. As such, we model them using ordered categorical regression with a linear model for ξ, and a vector of random cut-points C:

$$Y_{[i]} \sim \text{Ordered Categorical}(\xi_{[i]}, C) \tag{15}$$

with all other values being comparable to the omniscience model.

5 Analyses

All analyses are implemented using multi-level Bayesian regression models [5], with outcome distributions that are appropriately tailored to the empirical data; reproductive outcomes are fit using negative binomial regressions [3], ordered categorical outcomes are fit using ordered logistic regressions [6, 7], and interval constrained outcomes are fit using beta regressions [8]. For the main regression models, we focus on within-group variation by placing weak priors on the parameters controlling inter-site variation in intercepts and slopes (partial pooling). In the supplementary materials, we also report results for fully pooled models where inter-site variation was fixed at zero, strictly for the sake of comparison. All reported results include 90% equal tail posterior credibility intervals. Model descriptions, results tables, and additional analyses are included in the Supplementary Materials.

Data analysis was handled entirely in R (version 3.3.1) [9]. Statistical models were coded in Stan and fit using the RStan package (version 2.14.1) [10]. We diagnosed model fits and Markov Chain Monte Carlo performance using trace plots, $\hat{r}$, and reported effective samples [11]. Code for diagnostics and analysis replication are provided in the Supplementary R files.

5.1 Main analyses

The main analyses are reported in Tables D-G. The top blocks of these tables report the results for the moralistic deity models and the bottom blocks report the results for the local deity models. Variables denoted with "MD" in local deity models indicate the corresponding variable for moralistic deities.

Table D details models predicting attributed moral concern to deities. For the moralistic deity outcomes, the qualitative effects presented here are robust to analysis using a fully pooled model in Table I. The only strong effect found was the inclusion of moralistic deities. Table E reports the models for how much people claimed their deities know, Table F shows the supernatural punishment models, and Table G reports the ritual frequency models.

Age trends towards a positive effect on all four dependent variables for moralistic gods; older participants tend to claim moralistic deities know and punish more, claim these deities care more about morality, and claim to engage in rituals devoted to them more often. Note, however, that of these four religiosity models, all credibility intervals cross zero and the largest lower-bound of any credibility interval for age is -0.08 (for attributed moral concern). The same is not the case for local deities, where the credibility intervals for age on all four target variables are effectively symmetrical around zero.

Results from these analyses are qualitatively consistent with those produced in standard, non-Bayesian analyses using sites as varying effects. For instance, assessing Model 1 from Table 1 in the main text with a multilevel, zero-inflated negative binomial regression using field site as a higher-order variable shows that as simple effects, wealth (b = -0.11, 90% CI = [-0.23, 0.00]), education (b = -0.03, 90% CI = [-0.05, -0.01]), and sex (b = -0.15, 90% CI = [-0.26, -0.05]) maintain their negative relationship with number of children (intercept b = 0.04, 90% CI = [-0.23, 0.30], log-likelihood = -962.54). Code for this model is included in the scripts.

Table D. Cross-population mean estimates of outcomes of moral concern attributed to deities with 90% credibility intervals. M1 is the full model, and M2 and M3 drop the covariates of education and children in family, respectively. M4 in the local deity model adds a control for the moralization measure of the moralizing deity (MD). The top block of outcomes are the model results for the first outcome (beliefs about moralistic deities) and the bottom block of outcomes are the model results for the second outcome (beliefs about local deities). Note that the Local Deity models do not include the Lovu, Fiji sample as they did not answer these questions. Across populations, we see that none of the included variables are reliable predictors of belief in more moralistic deities. *Denotes credibility intervals that do not cross zero.

	Deity	M1	M2	M3	M4
Food Security	Moralistic	-0.03 (-0.29, 0.23)	-0.03 (-0.28, 0.22)	0.01 (-0.27, 0.28)	—
Education	Moralistic	0.00 (-0.07, 0.06)	—	0.00 (-0.05, 0.05)	—
Children	Moralistic	-0.02 (-0.12, 0.11)	-0.02 (-0.13, 0.10)	—	—
Age	Moralistic	0.13 (-0.08, 0.37)	0.14 (-0.06, 0.37)	0.07 (-0.10, 0.24)	—
Male	Moralistic	-0.04 (-0.29, 0.24)	-0.01 (-0.28, 0.26)	-0.01 (-0.26, 0.24)	—
Intercept	Moralistic	0.47 (-0.23, 1.21)	0.40 (-0.10, 0.90)	0.50 (-0.18, 1.15)	—
Food Security	Local	0.05 (-0.24, 0.34)	0.05 (-0.23, 0.33)	0.05 (-0.23, 0.34)	0.05 (-0.20, 0.32)
Education	Local	-0.02 (-0.06, 0.03)	—	-0.02 (-0.07, 0.02)	—
Children	Local	0.04 (-0.10, 0.19)	0.04 (-0.10, 0.20)	—	—
Age	Local	0.06 (-0.16, 0.27)	0.05 (-0.17, 0.28)	0.09 (-0.16, 0.38)	0.08 (-0.10, 0.27)
Male	Local	0.12 (-0.25, 0.48)	0.12 (-0.28, 0.55)	0.11 (-0.27, 0.53)	0.10 (-0.21, 0.43)
Moralistic (MD)	Local	—	—	—	2.18 (1.00, 3.26)*
Intercept	Local	-0.30 (-1.26, 0.55)	-0.38 (-1.24, 0.45)	-0.21 (-1.00, 0.57)	-1.88 (-2.44, -1.34)*

Table E. Cross-population mean estimates of outcomes of knowledge breadth attributed to deities with 90% credibility intervals. M1 is the full model, and M2 and M3 drop the covariates of education and children in family, respectively. M4 in the local deity model adds a control for the omniscience measure of the moralizing deity (MD). The top block of outcomes are the model results for the first outcome (beliefs about moralistic deities) and the bottom block of outcomes are the model results for the second outcome (beliefs about local deities). Note that the Local Deity models do not include the Lovu, Fiji sample as they did not answer these questions. Across populations, we see that none of the included variables are reliable predictors of the breadth of knowledge attributed to deities.

	Deity	M1	M2	M3	M4
Food Security	Moralistic	0.14 (-0.63, 1.08)	0.14 (-0.68, 1.08)	0.13 (-0.62, 1.05)	—
Education	Moralistic	0.02 (-0.14, 0.22)	—	0.02 (-0.13, 0.21)	—
Children	Moralistic	0.01 (-0.20, 0.23)	0.01 (-0.21, 0.23)	—	—
Age	Moralistic	0.39 (-0.21, 1.06)	0.46 (-0.18, 1.23)	0.40 (-0.12, 0.99)	—
Male	Moralistic	-0.11 (-0.90, 0.70)	-0.05 (-0.81, 0.75)	-0.10 (-0.84, 0.69)	—
Intercept	Moralistic	-0.02 (-7.92, 8.10)	0.04 (-8.25, 8.64)	0.02 (-8.47, 7.77)	—
Food Security	Local	-0.19 (-0.82, 0.40)	-0.18 (-0.74, 0.39)	-0.12 (-0.74, 0.50)	-0.14 (-0.88, 0.62)
Education	Local	0.00 (-0.17, 0.12)	—	0.00 (-0.21, 0.14)	—
Children	Local	-0.07 (-0.26, 0.15)	-0.09 (-0.26, 0.09)	—	—
Age	Local	0.09 (-0.74, 0.79)	-0.02 (-0.92, 0.74)	-0.07 (-0.65, 0.37)	-0.14 (-0.68, 0.31)
Male	Local	0.24 (-0.37, 0.81)	0.26 (-0.28, 0.82)	0.29 (-0.33, 0.90)	0.39 (-0.17, 1.02)
Knowledge (MD)	Local	—	—	—	1.31 (-0.03, 2.44)
Intercept	Local	0.10 (-7.75, 8.11)	-0.03 (-8.64, 8.68)	0.12 (-8.08, 7.74)	-0.06 (-8.19, 8.08)

Table F. Cross-population mean estimates of outcomes of punitiveness attributed to deities with 90% credibility intervals. M1 is the full model, and M2 and M3 drop the covariates of education and children in family, respectively. M4 in the local deity model adds a control for the punitiveness measure of the moralizing deity (MD). The top block of outcomes are the model results for the first outcome (beliefs about moralistic deities) and the bottom block of outcomes are the model results for the second outcome (beliefs about local deities). Note that the Local Deity models do not include the Lovu, Fiji sample as they did not answer these questions. Across populations, we see that none of the demographic variables are reliable predictors of respondents' claims about deities' punishment. *Denotes credibility intervals that do not cross zero.

	Deity	M1	M2	M3	M4
Food Security	Moralistic	0.14 (-0.37, 0.68)	0.15 (-0.32, 0.63)	0.14 (-0.38, 0.67)	—
Education	Moralistic	-0.02 (-0.08, 0.04)	—	-0.02 (-0.08, 0.04)	—
Children	Moralistic	-0.06 (-0.18, 0.05)	-0.05 (-0.17, 0.05)	—	—
Age	Moralistic	0.20 (-0.11, 0.52)	0.18 (-0.12, 0.52)	0.08 (-0.18, 0.33)	—
Male	Moralistic	0.04 (-0.37, 0.45)	0.03 (-0.38, 0.43)	0.05 (-0.35, 0.42)	—
Intercept	Moralistic	0.38 (-7.86, 9.01)	0.13 (-8.32, 8.62)	0.05 (-8.29, 7.96)	—
Food Security	Local	-0.14 (-0.62, 0.32)	-0.16 (-0.66, 0.32)	-0.17 (-0.70, 0.24)	-0.30 (-0.94, 0.28)
Education	Local	0.02 (-0.05, 0.10)	—	0.02 (-0.06, 0.09)	—
Children	Local	0.03 (-0.10, 0.14)	0.02 (-0.10, 0.13)	—	—
Age	Local	0.00 (-0.34, 0.34)	0.01 (-0.34, 0.36)	0.05 (-0.21, 0.30)	-0.08 (-0.37, 0.20)
Male	Local	0.45 (-0.23, 1.15)	0.46 (-0.22, 1.16)	0.44 (-0.29, 1.21)	0.32 (-0.23, 0.93)
Punishment (MD)	Local	—	—	—	1.24 (0.03, 2.41)*
Intercept	Local	-0.05 (-7.97, 7.43)	-0.16 (-8.70, 7.87)	0.33 (-7.37, 8.02)	-0.10 (-8.33, 8.19)

5.2 Supplementary analyses

Recall that Baumard et al.'s test [12] relies on point estimates for geographic regions and correlates them with other group-level factors. The *theory*, however, explicitly appeals to individual-level, life history patterns for which our models and main analyses in Tables D-G appropriately account. While Figure 2 in the main text suggests a linear trend at the group level, appropriately modeled data show no such trend. This should not be suprising given the precedent literature illustrating that using group-level data to address individual-level questions is fraught with inferential problems [13, 14].

To further illustrate the problem of not accounting for important variation, we also

Table G. Cross-population mean estimates of outcomes of ritual behavior with 90% credibility intervals. M1 is the full model, and M2 and M3 drop the covariates of education and children in family, respectively. M4 in the local deity model adds a control for the measure of ritual service dedicated to the moralizing deity (MD). The top block of outcomes are the model results for the first outcome (rituals devoted to moralistic deities) and the bottom block of outcomes are the model results for the second outcome (rituals devoted to local deities). Note that the Local Deity models do not include the Lovu, Fiji sample as they did not answer these questions. Across populations, we see that none of the demographic variables are reliable predictors of engaging in rituals devoted to deities.

	Deity	M1	M2	M3	M4
Food Security	Moralistic	0.26 (-0.19, 0.78)	0.25 (-0.22, 0.72)	0.22 (-0.23, 0.69)	—
Education	Moralistic	-0.01 (-0.09, 0.07)	—	-0.02 (-0.09, 0.06)	—
Children	Moralistic	0.05 (-0.10, 0.22)	0.05 (-0.09, 0.22)	—	—
Age	Moralistic	0.43 (-0.14, 1.02)	0.45 (-0.13, 1.10)	0.49 (-0.08, 1.06)	—
Male	Moralistic	0.13 (-0.33, 0.62)	0.14 (-0.36, 0.64)	0.14 (-0.34, 0.69)	—
Intercept	Moralistic	-0.20 (-8.68, 7.43)	-0.04 (-8.30, 8.44)	0.09 (-8.45, 8.49)	
Food Security	Local	-0.09 (-0.82, 0.63)	-0.16 (-0.87, 0.54)	-0.13 (-0.77, 0.59)	-0.23 (-0.97, 0.45)
Education	Local	0.05 (-0.15, 0.25)	—	0.03 (-0.18, 0.22)	—
Children	Local	0.11 (-0.13, 0.37)	0.10 (-0.15, 0.35)	—	—
Age	Local	0.24 (-0.88, 1.28)	0.11 (-1.20, 1.25)	0.40 (-0.77, 1.45)	0.15 (-0.87, 0.99)
Male	Local	-0.18 (-0.91, 0.55)	-0.12 (-0.74, 0.53)	-0.21 (-0.94, 0.52)	-0.21 (-0.84, 0.43)
Ritual (MD)	Local	—	—	—	0.36 (-0.06, 0.74)
Intercept	Local	-0.10 (-8.43, 7.82)	0.32 (-8.29, 8.44)	0.24 (-7.70, 8.46)	0.01 (-8.77, 8.02)

repeated the main analyses *without* modeling the hierarchical structure of the data (i.e., without modeling groups). Again, some results appear to be consistent with the theoretical predictions. After reporting the results from these fully-pooled models, we discuss the problems with drawing strong conclusions from them.

Table H reports the fully-pooled model for reproductive outcomes. The qualitative results are consistent with those found with the hierarchical models suggesting a trend both within and across the subsamples.

However, as reported in Table I, there are a few inconsistencies with those results reported in the main text. Number of children and food security appear to have a positive association with attribution of moral concern to local deities despite there being no other obvious associations between these religion variables and number of children (see Supplementary Materials in [1] for the correlation matrix of these variables). Recall the relationship between group-average material security and moralizing the local deities illustrated in Figure 2 in the main text.

Table J reports the results for gods' attributed knowledge breadth. For moralistic deities, education shows a negative relationship between gods' attributed knowledge breadth. Additionally, number of children show a positive relationship with the breadth of knowledge people claim their local deities have. Similarly, as reported in Table K, number of children and years of formal education predict increased punishment ratings for local deities. Table L reports the results for ritual performance frequencies. In this case, education appears to predict lower ritual frequency for both deities. However, number of children predicts *greater* self-reported frequency of rituals devoted to both deities.

6 Discussion

Some of these qualitative results appear to be consistent with Baumard et al.'s predictions [12, 15] and some suggest their reverse. As key demographic features of individuals change, so too do the kinds of features associated with the "Axial" religious traditions. However, it would be premature to conclude much from these results for at least three key reasons.

First, our main analyses model the data using random slopes and intercepts [5, 16]. In these supplementary analyses, we simply ignore within-group structure. This approach therefore assumes that the impact of material security—and conversely material *insecurity*—for example, is effectively the same across groups.

Second, ignoring groups is inconsistent with Baumard et al.'s [15] theoretical *predictions* which state that within populations, individuals with increased material security adopt slower life histories and more moralizing deities. As such, these predictions are best tested using models that estimate effects within populations. The fully pooled models do not do this. However, they do suggest that there may be some cross-cultural structuring linking material security and belief in more moralizing deities. There are innumerate missing variables which might explain this trend, so there is little room for causal inference.

A third possibility accounting for the discrepancies between fully and partially pooled analyses may stem from what's often referred to as "Simpson's paradox" [14] where the presence or direction of an effect can appear to change when considering group structure or some other confound. This being said, a wider cross-cultural study would be valuable for future theorizing.

Table H. Mean estimates and 90% credibility intervals of reproductive success outcomes for fully-pooled sample. M1 is the full model, and M2 and M3 drop education and food security outcomes, respectively. Across populations, we see proportionality between exposure time to risk of reproduction and reproductive success, as indicated by the elasticity estimate on age being centered on the value of 1. Males show reduced age-specific production of offspring relative to females. We note reliably negative average effects of education and wealth security on reproductive success. *Denotes credibility intervals that do not cross zero.

	M1	M2	M3
Food Security	-0.05	-0.09	—
	(-0.15, 0.05)	(-0.19, 0.02)	
Education	-0.05	—	-0.05
	(-0.06, -0.04)*		(-0.06, -0.04)*
Age (Elasticity)	0.95	1.02	0.94
	(0.86, 1.04)*	(0.93, 1.12)*	(0.86, 1.03)*
Male	-0.16	-0.12	-0.17
	(-0.26, -0.06)*	(-0.22, -0.02)*	(-0.27, -0.07)*
Intercept	-1.60	-2.18	-1.60
	(-1.94, -1.30)*	(-2.50, -1.87)*	(-1.92, -1.31)*

Table I. Mean estimates and 90% credibility intervals of the degree to which participants claim deities care about morality for fully-pooled sample. M1 is the full model, and M2 and M3 drop the covariates of education and children in family, respectively. M4 in the local deity model adds a control for the moralization measure of the moralistic deity (MD). The top block of outcomes are the model results for the first outcome (beliefs about moralistic deities) and the bottom block of outcomes are the model results for the second outcome (beliefs about local deities). *Denotes credibility intervals that do not cross zero.

	Deity	M1	M2	M3	M4
Food Security	Moralistic	-0.09 (-0.28, 0.09)	-0.09 (-0.26, 0.08)	-0.09 (-0.25, 0.08)	—
Education	Moralistic	0.00 (-0.02, 0.02)	—	0.00 (-0.02, 0.02)	—
Children	Moralistic	-0.02 (-0.07, 0.03)	-0.02 (-0.07, 0.03)	—	—
Age (Elasticity)	Moralistic	0.12 (-0.02, 0.26)	0.13 (-0.02, 0.27)	0.10 (-0.02, 0.21)	—
Male	Moralistic	-0.14 (-0.31, 0.04)	-0.13 (-0.29, 0.04)	-0.13 (-0.32, 0.06)	—
Intercept	Moralistic	0.43 (0.01, 0.83)*	0.40 (0.05, 0.76)*	0.44 (0.02, 0.87)*	—
Food Security	Local	0.30 (0.09, 0.52)*	0.30 (0.08, 0.52)*	0.27 (0.05, 0.48)*	0.33 (0.11, 0.56)*
Education	Local	0.01 (-0.02, 0.03)	—	0.00 (-0.02, 0.02)	—
Children	Local	0.08 (0.01, 0.15)*	0.07 (0.01, 0.14)*	—	—
Age (Elasticity)	Local	-0.12 (-0.30, 0.05)	-0.12 (-0.29, 0.06)	0.02 (-0.10, 0.15)	0.03 (-0.10, 0.15)
Male	Local	-0.09 (-0.32, 0.15)	-0.09 (-0.30, 0.11)	-0.13 (-0.34, 0.08)	-0.07 (-0.28, 0.13)
Moral Concern (MD)	Local	—	—	—	1.39 (1.02, 1.75)*
Intercept	Local	-0.21 (-0.65, 0.25)	-0.15 (-0.62, 0.30)	-0.3 (-0.75, 0.18)	-1.32 (-1.77, -0.85)*

Table J. Mean estimates and 90% credibility intervals of how much participants claim deities know for fully-pooled sample. M1 is the full model, and M2 and M3 drop the covariates of education and children in family, respectively. M4 in the local deity model adds a control for the knowledge measure of the moralistic deity (MD). The top block of outcomes are the model results for the first outcome (beliefs about moralistic deities) and the bottom block of outcomes are the model results for the second outcome (beliefs about local deities). *Denotes credibility intervals that do not cross zero.

	Deity	M1	M2	M3	M4
Food Security	Moralistic	-0.16 (-0.65, 0.35)	-0.18 (-0.67, 0.32)	-0.15 (-0.68, 0.37)	—
Education	Moralistic	-0.04 (-0.09, 0.01)	—	-0.02 (-0.07, 0.02)	—
Children	Moralistic	-0.09 (-0.24, 0.05)	-0.06 (-0.20, 0.08)	—	—
Age (Elasticity)	Moralistic	0.31 (-0.07, 0.70)	0.30 (-0.12, 0.69)	0.17 (-0.15, 0.47)	—
Male	Moralistic	-0.21 (-0.72, 0.33)	-0.16 (-0.64, 0.29)	-0.18 (-0.66, 0.30)	—
Intercept	Moralistic	0.09 (-7.70, 8.12)	0.01 (-8.00, 8.32)	0.11 (-8.29, 8.53)	—
Food Security	Local	0.01 (-0.35, 0.36)	0.00 (-0.29, 0.32)	-0.04 (-0.34, 0.24)	-0.04 (-0.34, 0.27)
Education	Local	0.00 (-0.04, 0.03)	—	-0.02 (-0.05, 0.01)	—
Children	Local	0.11 (0.03, 0.21)*	0.12 (0.04, 0.20)*	—	—
Age (Elasticity)	Local	-0.27 (-0.55, -0.01)*	-0.28 (-0.54, -0.02)*	-0.06 (-0.26, 0.14)	-0.08 (-0.29, 0.13)
Male	Local	0.06 (-0.27, 0.38)	0.07 (-0.25, 0.38)	0.00 (-0.33, 0.32)	0.01 (-0.30, 0.31)
Knowledge (MD)	Local	—	—	—	0.79 (0.48, 1.12)*
Intercept	Local	-0.19 (-8.27, 7.80)	0.13 (-7.85, 8.01)	0.20 (-7.79, 7.73)	0.28 (-7.62, 8.56)

Table K. Mean estimates and 90% credibility intervals of the degree to which participants claim deities punish people for fully-pooled sample. M1 is the full model, and M2 and M3 drop the covariates of education and children in family, respectively. M4 in the local deity model adds a control for the punishment measure of the moralistic deity (MD). The top block of outcomes are the model results for the first outcome (beliefs about moralistic deities) and the bottom block of outcomes are the model results for the second outcome (beliefs about local deities). *Denotes credibility intervals that do not cross zero.

	Deity	M1	M2	M3	M4
Food Security	Moralistic	0.00 (-0.29, 0.30)	0.00 (-0.28, 0.28)	0.00 (-0.27, 0.28)	—
Education	Moralistic	0.00 (-0.03, 0.03)	—	0.00 (-0.03, 0.03)	—
Children	Moralistic	-0.03 (-0.11, 0.05)	-0.03 (-0.10, 0.05)	—	—
Age (Elasticity)	Moralistic	0.14 (-0.07, 0.36)	0.14 (-0.08, 0.36)	0.09 (-0.09, 0.27)	—
Male	Moralistic	-0.09 (-0.38, 0.19)	-0.09 (-0.37, 0.20)	-0.08 (-0.36, 0.20)	—
Intercept	Moralistic	-0.17 (-8.53, 7.78)	-0.20 (-8.11, 7.31)	-0.15 (-7.98, 8.11)	—
Food Security	Local	0.05 (-0.25, 0.35)	0.02 (-0.29, 0.34)	0.00 (-0.30, 0.31)	-0.10 (-0.40, 0.20)
Education	Local	0.04 (0.01, 0.08)*	—	0.02 (0.00, 0.05)*	—
Children	Local	0.13 (0.03, 0.22)*	0.08 (0.00, 0.17)*	—	—
Age (Elasticity)	Local	-0.20 (-0.46, 0.08)	-0.15 (-0.42, 0.10)	0.05 (-0.15, 0.25)	0.03 (-0.19, 0.24)
Male	Local	0.10 (-0.21, 0.44)	0.04 (-0.28, 0.34)	0.04 (-0.25, 0.34)	0.01 (-0.33, 0.33)
Punishment (MD)	Local	—	—	—	1.10 (0.84, 1.37)*
Intercept	Local	0.03 (-8.21, 8.41)	0.11 (-8.11, 8.12)	-0.07 (-7.77, 8.30)	0.15 (-7.83, 8.11)

Table L. Mean estimates and 90% credibility intervals of the self-reported frequency of ritual devotions to deities for fully-pooled sample. M1 is the full model, and M2 and M3 drop the covariates of education and children in family, respectively. M4 in the local deity model adds a control for the ritual measure to the moralistic deity (MD). The top block of outcomes are the model results for the first outcome (beliefs about moralistic deities) and the bottom block of outcomes are the model results for the second outcome (beliefs about local deities). *Denotes credibility intervals that do not cross zero.

	Deity	M1	M2	M3	M4
Food Security	Moralistic	0.67 (0.37, 0.99)*	0.64 (0.35, 0.93)*	0.66 (0.35, 0.95)*	—
Education	Moralistic	-0.07 (-0.10 -0.04)*	—	-0.09 (-0.12, -0.06)*	—
Children	Moralistic	0.19 (0.10, 0.29)*	0.25 (0.16, 0.35)*	—	—
Age (Elasticity)	Moralistic	-0.25 (-0.49, -0.02)*	-0.27 (-0.52, -0.05)*	0.04 (-0.14, 0.22)	—
Male	Moralistic	0.33 (0.02, 0.65)*	0.41 (0.13, 0.70)*	0.24 (-0.06, 0.54)	—
Intercept	Moralistic	-0.20 (-8.50, 8.70)	-0.12 (-8.03, 8.21)	0.08 (-8.30, 8.25)	—
Food Security	Local	-0.04 (-0.43, 0.35)	-0.05 (-0.43, 0.34)	-0.10 (-0.48, 0.25)	-0.33 (-0.74, 0.08)
Education	Local	-0.07 (-0.11, -0.03)*	—	-0.10 (-0.14, -0.06)*	—
Children	Local	0.20 (0.09, 0.32)*	0.28 (0.17, 0.38)*	—	—
Age (Elasticity)	Local	-0.12 (-0.43, 0.18)	-0.21 (-0.52, 0.08)	0.24 (0.02, 0.46)*	0.19 (-0.05, 0.43)
Male	Local	-0.29 (-0.65, 0.06)	-0.14 (-0.54, 0.24)	-0.41 (-0.78, -0.05)*	-0.46 (-0.87, -0.07)*
Ritual (MD)	Local	—	—	—	0.97 (0.80, 1.16)*
Intercept	Local	-0.08 (-8.24, 8.20)	-0.32 (-8.05, 7.15)	-0.17 (-8.15, 7.52)	-0.13 (-8.50, 7.93)

References

1. Purzycki BG, Apicella C, Atkinson QD, Cohen E, McNamara RA, Willard AK, et al. Moralistic gods, supernatural punishment and the expansion of human sociality. Nature. 2016;530(7590):327–330. doi:10.1038/nature16980.

2. Purzycki BG, Apicella C, Atkinson QD, Cohen E, McNamara RA, Willard AK, et al. Cross-cultural dataset for the evolution of religion and morality project. Scientific Data. 2016;3:160099. doi:10.1038/sdata.2016.99.

3. Oh SY, Ross, C., Borgerhoff Mulder, M, Bowles, S. The decline of polygyny: an interpretation. Santa Fe Institute Working Paper. 2016. Available from: https://sfi-edu.s3.amazonaws.com/sfi-edu/production/uploads/working_paper/pdf/2017-12-037_ae5724.pdf.

4. Hruschka D, Efferson C, Jiang T, Falletta-Cowden A, Sigurdsson S, McNamara R, et al. Impartial institutions, pathogen stress and the expanding social network. Human Nature. 2014;25(4):567–579. doi:10.1007/s12110-014-9217-0.

5. McElreath R. Statistical Rethinking: A Bayesian Course with Examples in R and Stan. vol. 122. CRC Press; 2016.

6. McCullagh P. Regression models for ordinal data. Journal of the Royal Statistical Society Series B (Methodological). 1980;42(2):109–142.

7. Ross CT, Winterhalder B. A hierarchical bayesian analysis of parasite prevalence and sociocultural outcomes: The role of structural racism and sanitation infrastructure. American Journal of Human Biology. 2016;28(1):74–89.

8. Ross CT. Sliding-scale environmental service payments and non-financial incentives: Results of a survey of landowner interest in Costa Rica. Ecological Economics. 2016;130:252 – 262. doi:http://dx.doi.org/10.1016/j.ecolecon.2016.07.014.

9. R Core Team. R: A Language and Environment for Statistical Computing; 2016. Available from: https://www.R-project.org/.

10. Stan Development Team. Stan: A C++ Library for Probability and Sampling, Version 2.10.0; 2015. Available from: http://mc-stan.org/.

11. Gelman A, Carlin JB, Stern HS, Dunson DB, Vehtari A, Rubin DB. Bayesian Data Analysis. CRC Press; 2014.

12. Baumard N, Hyafil A, Morris I, Boyer P. Increased affluence explains the emergence of ascetic wisdoms and moralizing religions. Current Biology. 2015;25(1):10–15. doi:10.1016/j.cub.2014.10.063.

13. Robinson WS. Ecological correlations and the behavior of individuals. American Sociological Review. 1950;15(3):351–357.

14. Simpson EH. The interpretation of interaction in contingency tables. Journal of the Royal Statistical Society Series B (Methodological). 1951;13(2):238–241.

15. Baumard N, Chevallier C. The nature and dynamics of world religions: a life-history approach. Proceedings of the Royal Society B: Biological Sciences. 2015;282(1818):20151593. doi:10.1098/rspb.2015.1593.

16. Gelman A, Hill J. Data Analysis Using Regression and Multilevel/Hierarchical Models. Cambridge University Press; 2006.
